# Supplementary material for: Inferring differential subcellular localisation in comparative spatial proteomics using BANDLE
Source: Nat Commun. 2022 Oct 10;13:5948. doi: 10.1038/s41467-022-33570-9 (PMC9550814; doi:10.1038/s41467-022-33570-9)
Supplement: Supplementary file 3 — Reporting Summary [file 41467_2022_33570_MOESM3_ESM.pdf]

nature portfolio

Corresponding author(s): Oliver Crook

Last updated by author(s): 02/09/2022

Reporting Summary

Nature Portfolio wishes to improve the reproducibility of the work that we publish. This form provides structure for consistency and transparency in reporting. For further information on Nature Portfolio policies, see our Editorial Policies and the Editorial Policy Checklist.

Statistics

For all statistical analyses, confirm that the following items are present in the figure legend, table legend, main text, or Methods section.

n/a	Confirmed
☐	☒ The exact sample size (*n*) for each experimental group/condition, given as a discrete number and unit of measurement
☐	☒ A statement on whether measurements were taken from distinct samples or whether the same sample was measured repeatedly
☐	☒ The statistical test(s) used AND whether they are one- or two-sided<br>*Only common tests should be described solely by name; describe more complex techniques in the Methods section.*
☐	☒ A description of all covariates tested
☐	☒ A description of any assumptions or corrections, such as tests of normality and adjustment for multiple comparisons
☐	☒ A full description of the statistical parameters including central tendency (e.g. means) or other basic estimates (e.g. regression coefficient) AND variation (e.g. standard deviation) or associated estimates of uncertainty (e.g. confidence intervals)
☐	☒ For null hypothesis testing, the test statistic (e.g. *F*, *t*, *r*) with confidence intervals, effect sizes, degrees of freedom and *P* value noted<br>*Give P values as exact values whenever suitable.*
☐	☒ For Bayesian analysis, information on the choice of priors and Markov chain Monte Carlo settings
☐	☒ For hierarchical and complex designs, identification of the appropriate level for tests and full reporting of outcomes
☐	☒ Estimates of effect sizes (e.g. Cohen's *d*, Pearson's *r*), indicating how they were calculated

Our web collection on statistics for biologists contains articles on many of the points above.

Software and code

Policy information about availability of computer code

Data collection	R version 4.1 was used to collect the data. String version 11.5 was used to collect annotation and pathway enrichment results.
Data analysis	R version 4.1 was used to collect the data. The bandle package version 1.0 was used to analyse the data, along with custom code provided in the zenodo repository. The following R package were used MASS_7.3-54, patchwork_1.1.1, reshape2_1.4.4, ggplot2_3.3.5, pRolocdata_1.33.1, pRoloc_1.33.0, BiocParallel_1.27.16, MLInterfaces_1.72.0, cluster_2.1.2, annotate_1.70.0, XML_3.99-0.8, AnnotationDbi_1.56.2, IRanges_2.26.0, MSnbase_2.18.0, ProtGenerics_1.25.1, mzR_2.26.1, Rcpp_1.0.7, Biobase_2.52.0 S4Vectors_0.30.2, BiocGenerics_0.40.0

For manuscripts utilizing custom algorithms or software that are central to the research but not yet described in published literature, software must be made available to editors and reviewers. We strongly encourage code deposition in a community repository (e.g. GitHub). See the Nature Portfolio guidelines for submitting code & software for further information.

Data

Policy information about availability of data

All manuscripts must include a data availability statement. This statement should provide the following information, where applicable:

- Accession codes, unique identifiers, or web links for publicly available datasets
- A description of any restrictions on data availability
- For clinical datasets or third party data, please ensure that the statement adheres to our policy

The spatial proteomics data has been deposited and is available in the Bioconductor package pRolocdata. The additional data are given in the referenced

manuscripts and additionally are provided as part of the supplementary material. The MCMC data generated in this study has been deposited in Zenodo: \url{https://doi.org/10.5281/zenodo.4415369}, supplementary code and data are deposited at Zenodo: \url{https://doi.org/10.5281/zenodo.6514300}. The AP-4 dataset (PXD010103) is available at \url{http://proteomecentral.proteomexchange.org/cgi/GetDataset?ID=PXD010103}. The HCMV spatial protemics dataset (PXD003925) is available at \url{http://proteomecentral.proteomexchange.org/cgi/GetDataset?ID=PXD003925.}. The HCMV acetylation dataset (PXD009839) is available at \url{http://proteomecentral.proteomexchange.org/cgi/GetDataset?ID=PXD009839}. The HCMV degredation assays datasets (PXD009945) are available at \url{http://proteomecentral.proteomexchange.org/cgi/GetDataset?ID=PXD009945}. The HCMV interactome dataset (PXD014845) is available at \url{http://proteomecentral.proteomexchange.org/cgi/GetDataset?ID=PXD014845}. String version 11.5 database was used to retrieve annotations and pathway enrichment results (https://string-db.org/)

Human research participants

Policy information about studies involving human research participants and Sex and Gender in Research.

Reporting on sex and gender	n/a
Population characteristics	n/a
Recruitment	n/a
Ethics oversight	n/a

Note that full information on the approval of the study protocol must also be provided in the manuscript.

Field-specific reporting

Please select the one below that is the best fit for your research. If you are not sure, read the appropriate sections before making your selection.

[x] Life sciences [ ] Behavioural & social sciences [ ] Ecological, evolutionary & environmental sciences

For a reference copy of the document with all sections, see nature.com/documents/nr-reporting-summary-flat.pdf

Life sciences study design

All studies must disclose on these points even when the disclosure is negative.

Sample size	Simulations are presented over 10 datasets in each scenario. This allowed the comparisons of statistics between competing methods. 3 experimental datasets were available at the time and represent the full spectrum of experimental designs in spatial proteomics.
Data exclusions	Classes were the markers were further from each other than to other classes were excluded.
Replication	Simulations always generate 3 replicates. The EGF experiment was presented in triplicates, the AP-4 dataset was replicated (n = 2), the HCMV spatial proteomics dataset was not replicated as it was derived from a time-course experiment. All other proteomics dataset is from 3 replicates.
Randomization	Randomization is not relevant, since this is not a populations level analysis to study different treatment groups.
Blinding	Blinding is not relevant, since it is impossible to perform the analysis with blinding.

Reporting for specific materials, systems and methods

We require information from authors about some types of materials, experimental systems and methods used in many studies. Here, indicate whether each material, system or method listed is relevant to your study. If you are not sure if a list item applies to your research, read the appropriate section before selecting a response.

Materials & experimental systems

n/a	Involved in the study
[x]	[ ] Antibodies
[x]	[ ] Eukaryotic cell lines
[x]	[ ] Palaeontology and archaeology
[x]	[ ] Animals and other organisms
[x]	[ ] Clinical data
[x]	[ ] Dual use research of concern

Methods

n/a	Involved in the study
[x]	[ ] ChIP-seq
[x]	[ ] Flow cytometry
[x]	[ ] MRI-based neuroimaging
